# Differentiating treatment episodes from gaps in eyes with diabetic macular oedema

**DOI:** 10.1136/bjo-2025-327238

**Published:** 2025-07-01

**Authors:** Yohei Hashimoto, Adrian Robert Hunt, Rufino Silva, Antonella Witmer, Wajiha Jurdi Kheir, Francesco Viola, Maite Arrazola, Mohamed Moghazy Mahgoub, Andreas Pollreisz, Zanne Harvey, Daniel Barthelmes, Mark C Gillies

**Affiliations:** 1 Save Sight Institute, The University of Sydney, Sydney, New South Wales, Australia; 2 Ophthalmology, Westmead Hospital, Westmead, New South Wales, Australia; 3 Department of Ophthalmology, Hospital of the University of Coimbra, Coimbra, Portugal; 4 Department of Ophthalmology, OLVG Hospital, Amsterdam, The Netherlands; 5 Opthalmology, American University of Beirut Medical Center, Beirut, Lebanon; 6 Ophthalmology, Universita degli Studi di Milano, Milano, Italy; 7 Department of Ophthalmology, University Hospital of Basurto, Bilbao, Spain; 8 Ain Shams University, Cairo, Egypt; 9 Department of Ophthalmology, Medical University of Vienna, Vienna, Austria; 10 Eyes Wide Bay, Hervey Bay, Queensland, Australia; 11 Universitatsspital Zurich Augenklinik und Poliklinik, Zurich, Switzerland

**Keywords:** Macula, Treatment Medical

## Abstract

The treatment of diabetic macular oedema (DMO) likely involves a series of treatment episodes separated by gaps during which no vascular endothelial growth factor inhibitor injections are delivered. Our aim is to differentiate the episodes and gaps with the isolation forests algorithm using the Fight Retinal Blindness! registry. We analysed 11 786 injection intervals (12 803 injections) and found that the period between adjacent injections≥38 weeks (95% CI 34 to 43 weeks) apart could be regarded as a treatment gap. The results will allow treatment episodes to be isolated so that the treatment patterns of DMO can be characterised more accurately.

## Introduction

**Figure 1:**
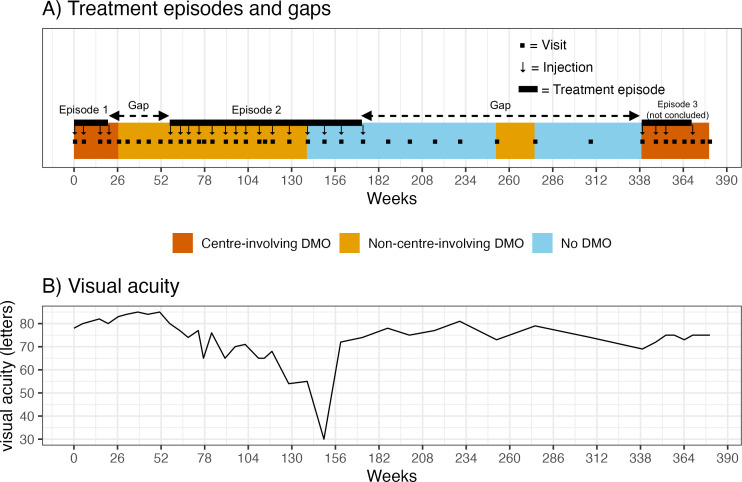
An example of treatment episodes and gaps with the trajectory of VA. (**A**) Treatment episodes and gaps in an eye. (**B**) The trajectory of VA for the same eye. DMO, diabetic macular oedema; VA, visual acuity.

 Practitioners frequently start treating diabetic macular oedema (DMO) with 4-weekly injections, but they expect to progressively reduce the treatment frequency and eventually attempt the cessation of treatment.^1^ Nevertheless, many eyes with DMO must resume treatment if their oedema recurs. Thus, the treatment of DMO can be regarded as a series of treatment ‘episodes’ separated by ‘gaps’ during which the eye is observed without treatment (figure 1). Differentiating these episodes from gaps would improve our understanding of treatment patterns in DMO. Injection intervals and treatment regimens (reactive or proactive) can be calculated more accurately by focusing on the episodes while excluding the gaps. Currently, there is no consensus on what period between injections constitutes a gap in the treatment of DMO.

What does make sense clinically is that, when transitioning from a treatment gap to a new treatment episode, the injection interval would significantly reduce. We will identify the transition using an outlier detection algorithm.

## Methods

### Data source

This is a retrospective cohort study using the Fight Retinal Blindness! registry designed to collect the real-world outcomes of retinal diseases worldwide.^2^ The details of the registry are described elsewhere.^2^ Participants from practices in Australia, France, Italy, the Netherlands, New Zealand, Portugal, Spain, Switzerland and the UK were included. The details of the ethics approval are written in online supplemental text.

### Eye selection

We included eyes with DMO in patients aged ≥20 years that started vascular endothelial growth factor (VEGF) inhibitor injections between January 2016 and January 2024. Only eyes that received aflibercept, bevacizumab or ranibizumab were included because brolucizumab, faricimab and intravitreal corticosteroid (dexamethasone, fluocinolone or triamcinolone) may have longer durability.^3^,^5^ Eyes that received ≥6 injections were included because enough injections were needed to detect a change in injection intervals. Eyes that had vitreous haemorrhage or vitrectomy were excluded because VEGF inhibitors might have been used to treat preretinal neovascularisation rather than DMO. Countries with<40 eyes were excluded to conduct the analyses stratified by country. The eyes were followed up from the first injection and censored at the last recorded injection.

### Statistical analysis

The time point at which the injection intervals decreased significantly was regarded as the beginning of the next treatment episode. Table 1 shows an example where the injection interval significantly changed from 5th to 6th injections and from 20th to 21st injections, after which a new treatment episode was likely to start. The injection intervals before the decrease (37 and 167 weeks) will be regarded as a gap in treatment.

**Table 1:** Injection history for an eye

Nth of injection	Injection date	Injection interval, weeks	Isolation forests score
1	23-01-2017		
2	01-03-2017	5	0.45
3	09-05-2017	10	0.44
4	15-06-2017	5	0.45
5	27-02-2018	37	0.73
6	10-04-2018	6	0.37
7	15-05-2018	5	0.54
8	26-06-2018	6	0.37
9	21-08-2018	8	0.37
10	16-10-2018	8	0.37
11	27-11-2018	6	0.37
12	08-01-2019	6	0.37
13	05-03-2019	8	0.37
14	30-04-2019	8	0.37
15	09-07-2019	10	0.35
16	24-09-2019	11	0.48
17	03-12-2019	10	0.35
18	11-02-2020	10	0.35
19	12-05-2020	13	0.53
20	25-07-2023	167	0.86
21	19-09-2023	8	0.37
22	31-10-2023	6	0.37
23	20-02-2024	16	0.59

This eye is the same as the one for figure 1. The injection interval significantly changed at the 5th and 20th injections, after which a new treatment episode was likely to start. These injection intervals (37 weeks and 167 weeks) will be regarded as a gap in treatment.

This significant change in the injection interval can be regarded as an outlier in the sequence of intervals *within* an eye (*local* outliers^6^). Isolation forests, an unsupervised machine learning algorithm, were applied to each eye to detect the outliers.^7^ The outlier score ranges between 0 and 1 where the scores closer to 1 are considered more likely to be outliers.^7^ The injection intervals where the outlier score was ≥0.7^7^ and the next interval decreased were regarded as treatment gaps.

We added a criterion of *global* outliers^6^ to improve outlier detection and gap identification. Online supplemental table 1 shows an example where the injection interval changed from 4 to 5 weeks at the 7th injection, leading to a *local* (within the treatment of one eye) isolation forests score of≥0.7, but it makes no sense to classify this interval as a treatment gap. Thus, a *global* comparison against *all* intervals was added with intervals above $third\;quartile\left(Q3\right)+1.5\;interquantile\;range\;\left(IQR\right)$ of all intervals regarded as global outliers.^8^ The outcome of interest was the median of the treatment gaps (ie, local and global outliers). The 95% CI was estimated using the non-parametric percentile bootstrap. The same analyses were conducted stratified by country.

## Results

A total of 11 786 intervals (median, 6 weeks (IQR, 4–9 weeks)) with 1017 eyes and 12 803 injections were eligible for analysis. Males accounted for 59% of eyes, mean baseline age was 64 years and mean follow-up period was 124 weeks. The isolation forests found that 495 intervals had an outlier score of ≥0.7 (local outliers). Of these intervals, 366 were greater than the threshold for global outliers (16 weeks for this study). The 366 (3.1% of all the intervals (366/11 786)) outliers were regarded as treatment gaps with a median (95% CI based on the bootstrapping) of 38 weeks (34–43 weeks) (table 2). The median of the treatment gaps by country was within a range of 22–47 weeks. Most of these treatment gaps (69%, 251/366) included visits at which treatment was not given.

**Table 2:** Treatment gaps by country

Country	Eyes, n	Injections, n	Median, weeks (95% CI)
All countries	1017	12 803	38 (34 to 43)
Australia	380	4907	47 (43 to 54)
France	155	2027	38 (28 to 45)
Netherlands	75	1111	22 (18 to 25)
New Zealand	121	1390	31 (25 to 45)
Spain	140	1611	34 (29 to 41)
Switzerland	46	790	37 (30 to 65)
UK	100	967	22 (20 to 31)

## Discussion

We found that adjacent injections≥38 weeks apart can be regarded as the end of one treatment episode and the beginning of the next with the period between them as a gap. This study was based on a scientific analysis, unlike previous studies that subjectively defined the treatment gap as 180^9^ or 365 days^10^ without VEGF inhibitors given.

Excluding the gap in treatment will allow us to accurately estimate the different treatment regimens (proactive/reactive) for DMO and their respective outcomes (figure 1). It will also become possible to investigate visual acuity, central subfield thickness and DMO activity within each treatment episode. Those analyses would provide more accurate information than those just reporting mean injection numbers and intervals over predetermined periods.

The treatment gap varied somewhat from one country to the next. Countries with shorter treatment gaps like the Netherlands tended to use fixed dosing regimens with shorter intervals, while countries like Australia with longer gaps tended to use a more proactive approach (data not shown). This will be explored closely in our next analysis on the outcomes of various treatment patterns in eyes with DMO.

Our study has several limitations. First, the treatment gap was decided without considering visual acuity or DMO activity. Future studies investigating these measurements after removing treatment gaps would support its validity. Second, there is no specific threshold of the outlier score above which a point is defined as an outlier. We followed the threshold used in a previous paper^7^ and the results seemed to be reasonable from a clinical perspective. Third, 31% of treatment gaps did not have visits recorded within them. We cannot say whether this was because patients missed visits or whether the practitioner had deliberately extended the follow-up period. We did not treat these cases differently because a certain interval between injections can be regarded as a treatment gap for the purposes of this study irrespective of the cause. Most gaps included visits without treatment, indicating that patients still seemed compliant with treatment.

Findings suggest that injections≥38 weeks apart can be regarded as the end and the beginning, respectively, of treatment episodes, with the time between the two as treatment gaps. Separating these episodes and gaps will improve the understanding of DMO treatment patterns and their respective safety and efficacy.

## Supplementary material

10.1136/bjo-2025-327238 online supplemental file 1

10.1136/bjo-2025-327238 online supplemental file 2

## Data Availability

No data are available.
