# Supplementary material for: Differentiating treatment episodes from gaps in eyes with diabetic macular oedema
Source: Br J Ophthalmol. 2025 Jul 1;109(11):e327238. doi: 10.1136/bjo-2025-327238 (PMC12573354; doi:10.1136/bjo-2025-327238)
Supplement: online supplemental file 1 [file bjo-109-11-s001.docx]

**Supplementary text**

In Australia, the Institutional ethics approval was obtained from the Sydney Local Health District Human Research Ethics, Western Sydney Local Human Research Ethics Committee and The Royal Australian and New Zealand College of Ophthalmologists Ethics Committee. Other countries listed in this paper were included after they obtained their own national ethics committee approvals. This research adhered to Australian National Statement on Ethical Conduct in Human Research guidelines.
