# Supplementary material for: Differentiating treatment episodes from gaps in eyes with diabetic macular oedema
Source: Br J Ophthalmol. 2025 Jul 1;109(11):e327238. doi: 10.1136/bjo-2025-327238 (PMC12573354; doi:10.1136/bjo-2025-327238)
Supplement: online supplemental file 2 [file bjo-109-11-s002.docx]

Supplementary Table 1: Injection history for an eye where isolation forests outlier scores are not sufficient

| Nth of injection | Injection date | Injection interval, weeks | Isolation forests score |
| --- | --- | --- | --- |
| 1 | 2021-02-19 |  |  |
| 2 | 2021-03-19 | 4 | 0.46 |
| 3 | 2021-04-16 | 4 | 0.46 |
| 4 | 2021-05-14 | 4 | 0.46 |
| 5 | 2021-06-11 | 4 | 0.46 |
| 6 | 2021-07-09 | 4 | 0.46 |
| 7 | 2021-08-10 | 5 | 0.74 |
| 8 | 2021-09-07 | 4 | 0.46 |
| 9 | 2021-10-05 | 4 | 0.46 |
| 10 | 2021-11-02 | 4 | 0.46 |
| The injection interval slightly increased from 4 weeks to 5 weeks at the 7th injection with the isolation forests score of ≥0.7. Since it made no sense to classify this case as a treatment gap, another criterion was necessary where treatment gaps should be global outliers against all intervals for the eyes. | | | |
